# Alzheimer disease is (sometimes) highly heritable: Drivers of variation in heritability estimates for binary traits, a systematic review

**DOI:** 10.1101/2025.04.29.25326648

**Published:** 2025-04-30

**Authors:** Shiying Liu, William S. Bush, Rufus Olusola Akinyemi, Goldie S. Byrd, Allison Mercedes Caban-Holt, Farid Rajabli, Christiane Reitz, Brian W. Kunkle, Giuseppe Tosto, Jeffery M. Vance, Margaret Pericak-Vance, Jonathan L. Haines, Scott M. Williams, Dana C. Crawford

**Affiliations:** 1. Department of Population and Quantitative Health Sciences, Case Western Reserve University School of Medicine, Cleveland, OH, USA; 2. Neuroscience and Ageing Research Unit, Institute for Advanced Medical Research and Training, College of Medicine, University of Ibadan, Ibadan, Oyo, Nigeria; 3. Maya Angelou Center for Health Equity, Wake Forest School of Medicine, Winston-Salem, NC, USA; 4. Department of Behavioral Science, College of Medicine and Sanders-Brown Center on Aging, University of Kentucky, Lexington, KY, USA; 5. John P. Hussman Institute for Human Genomics, Dr. John T. Macdonald Foundation Department of Human Genetics, Department of Neurology, University of Miami Miller School of Medicine, Miami, FL, USA; 6. Gertrude H. Sergievsky Center, Taub Institute for Research on Alzheimer’s Disease and the Aging Brain, Department of Neurology Columbia University, Department of Epidemiology, Columbia University, New York, NY, USA.; 7. The Taub Institute for Research on Alzheimer’s Disease and the Aging Brain, Columbia University Irving Medical Center, Columbia University; Department of Neurology, Columbia University Irving Medical Center, Columbia University; The Gertrude H. Sergievsky Center, College of Physicians and Surgeons, Columbia University Irving Medical Center, Columbia University, New York, NY USA.; 8. Dr. John T. Macdonald Foundation Department of Human Genetics, University of Miami Miller School of Medicine, Miami, Florida, USA

## Abstract

Estimating heritability has been fundamental in understanding the genetic contributions to complex disorders like late-onset Alzheimer’s disease (LOAD), and provides a rationale for identifying genetic factors associated with disease susceptibility. While numerous studies have established substantial genetic contribution for LOAD, the interpretation of heritability estimates remains challenging. These challenges are further complicated by the binary nature of LOAD status, where estimation and interpretation require additional considerations. Through a systematic review, we identified LOAD heritability estimates from 6 twin studies and 17 genome-wide association studies, all conducted in populations of European ancestry. We demonstrate that these heritability estimates for LOAD vary considerably. The variation reflects not only differences in study design and methodological approaches but also the underlying study population characteristics. Our findings indicate that commonly cited heritability estimates, often treated as universal values, should be interpreted within specific population contexts and methodological frameworks.

## Introduction

Heritability is commonly defined as the proportion of phenotypic variation attributable to genetics [1], a concept first described mathematically more than a century ago [2]. Early estimates of heritability for human traits were determined using family study design, while more contemporary estimates leverage population-scale studies with genome-wide genotyping or sequencing data [1,3,4]. Heritability estimates from the 1980s through the early 2000s were initially used to demonstrate that a trait of interest has a measurable genetic component [5], justifying the search for specific genes using either genetic linkage analyses or genetic association [6]. Almost 20 years into the era of genome-wide association studies (GWAS) and genome sequencing, estimates of heritability can now be used to estimate how much of a trait’s genetic component is yet to be discovered [7,8], thereby justifying additional genetic studies [9,10].

Both how heritability is estimated and how it is used have evolved over the past few decades. The literature on heritability study designs, statistical methods, and applications is deep, highlighting general strengths, weaknesses, and misinterpretations [3,4,11–13]. One major misinterpretation is that heritability is a fixed quantity [3,4]. In reality, heritability estimates are complex and dynamic, changing as a result of variable environmental effects and their interactions with genetic factors [14–17]. Study designs and statistical methodology also impact resulting heritability estimates. As an example, the heritability of the quantitative trait human height has been studied extensively for over a century. Classical family studies have consistently estimated the heritability of height at ~ 80% [18], while the large-scale genetic studies of unrelated participants estimated that imputed genetic variation accounts for 56% of the observed phenotypic variance [19], illustrating the ceiling and floor of heritability estimates possible when different study designs and methodologies are deployed on different datasets from different contexts and samples.

Methods to estimate heritability were originally developed for quantitative traits. The concept was then extended and applied to binary traits such as late-onset Alzheimer’s disease (LOAD), a complex neurodegenerative disorder characterized by memory loss and dementia among adults ≥65 years of age [20,21]. One of the most highly cited estimates presents a very high heritability for LOAD (79%) [22], and while the identification of LOAD-associated genetic variants and genes has been successful [23–27], the range of LOAD heritability across a variety of contexts and methodologies and its implications for the discovery of new associating genes are not yet fully appreciated. It is worth highlighting that the methods for binary outcomes, such as LOAD, require additional steps for heritability estimation and cautious interpretation as compared to those for quantitative traits. This latter point we argue is not thoroughly described in the literature. In this systematic review, we examine LOAD heritability estimates, illustrate differences across study populations and study designs, and highlight differences based on assumptions and methodologies employed. Overall, we found that published LOAD heritability estimates indicate that generalizability is limited. In addition, the variation in reported LOAD heritability reveals population or cohort-specific variability that may be important in both identifying LOAD loci and designing interventions among populations.

## Methods

In this systematic review, we focus on narrow sense (h^2^) heritability of LOAD as this is generally the measure presented in the literature. Heritability can also be estimated as broad-sense (H^2^) heritability. H^2^ captures the total genetic variance, which includes additive (A) and dominance (D) effects, as well as the epistatic effect (I). In contrast, h^2^ only defines the additive genetic component. While closely related, these two metrics describe distinct but overlapping aspects of genetic influence on phenotypic variation within a population. Distinguishing these forms is important, as each has different implications and requires flexible interpretation in research contexts [11,28].

### Data collection

Data were collected from the primary literature and included original, peer-reviewed studies of LOAD narrow sense heritability. Our primary literature search was conducted using PubMed, the National Library of Medicine’s free resource for the search and retrieval of citations and abstracts from MEDLINE and PubMed Central (PMC) databases [29]. We formulated the search query as “Alzheimer’s disease” AND “heritability,” tailored to PubMed’s search functions, to identify articles that mentioned heritability values for LOAD. This initial search, conducted in April 2024, yielded 624 articles, including both research and review papers.

In addition to the initial search, we explored other resources to ensure that our review was comprehensive and not limited by PubMed or the corresponding search terms we used. These included relevant publications identified from citations of published twin registries focusing on LOAD, along with citations within and publications citing the recent large LOAD GWAS captured in the initial search results. This “backward searching” added 52 articles, and these articles were combined with our initial search results and screened using the approaches as described below.

**Fig 1.**
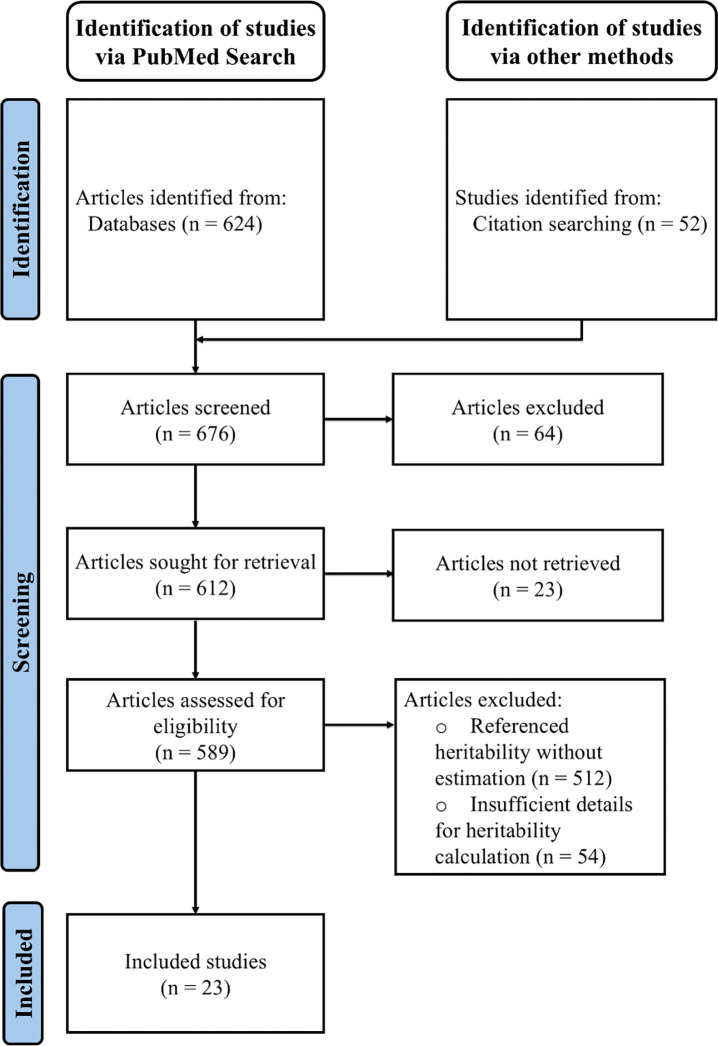
PRISMA flow diagram. Created following the PRIMSMA 2020 guidelines [32].

To finalize the pool of articles to include in this systematic review, we applied a multi-step filtering process to identify relevant studies from the initial search, excluding studies that only mention or restate published heritability values. Manual screening of the titles and abstracts of all 676 papers was first performed to exclude articles that were irrelevant, such as those not focusing on LOAD. This initial screening eliminated 64 papers, leaving 612 papers to consider. Of these 612, 23 were excluded because they could not be retrieved through institutional access or were non-English publications. For the remaining 589 articles, we conducted a comprehensive screening of both abstracts and full texts to identify studies that involved original research on the heritability of LOAD. We first removed studies that only referenced the heritability value(s) of LOAD without calculating their own estimates (n=512; Fig 1). We then performed an in-depth full-text examination of the remaining 77 papers to determine if they met our criteria for conducting original research on heritability estimation for LOAD. We defined original research as studies with detailed methods for computing heritability, including details of the study design, modeling process, and study population. Manuscripts without sufficient detail were excluded from further consideration. For example, one retrieved twin study lacked explicit details on how the LOAD heritability estimates were derived [30]. A parent-offspring study was also excluded due to inaccessible supplementary materials that described both the study population and the detailed methodology [31]. In total, 54 manuscripts were excluded at this step (Fig 1), leaving 23 peer-reviewed manuscripts with sufficient detail for this review.

### Characteristics of the Literature

We categorized the 23 eligible studies based on the study design and methods employed. The evaluation included the data sources, the study design employed in the original studies, the assumptions embedded in each approach, and the populations to which these estimates are intended to be generalized [8,19,28,33–36]. Heritability estimation methods can be categorized into two primary groups: 1) family-based methods, rooted in studies of related individuals, such as twins and extended pedigrees, without necessarily requiring genomic data, and 2) single nucleotide polymorphism (SNP)-based methods, consisting of population-based methods leveraging genome-wide data from large samples of often unrelated individuals. Based on this, we examined the articles, focusing on their methodologies, and stratified them into twin-based and SNP-based heritability estimates. Among the 23 articles passing the criteria, seven were based on twin studies, while 16 were SNP based studies.

This review has not been registered, and no protocol was prepared.

## Results

We identified a total of 23 relevant studies published between 1997 and 2023, including both twin-based and SNP-based studies. In addition to the heritability estimate and standard error, for each study the following essential information was collected for all studies: study population characteristics, sample size, case criteria, and specific estimation methods (Tables 1–3). For SNP-based studies, we also collected number of SNPs, included covariates, summary statistics dataset reference, and prevalence used for liability transformation (Tables 2 and 3). The heritability estimates for LOAD across different study designs and populations ranged from 3.1% [26] to 79% [22]. These studies primarily focused on participants of European ancestry, with a mean age mostly greater than 70 years, except for one study leveraging the National Research Council Registry of Aging Twin Veterans that has an average age of 63.1 years [37]. The sample sizes varied considerably, ranging from 38 twin pairs [38] to 761,704 participants in a SNP-based study [26]. Compared with SNP-based estimates, heritability estimates from family-based studies generally provided higher values, representing the likely ceiling of trait heritability [8]. SNP-based estimates correspond to the proportion of genetic variance explained by SNPs assayed, imputed, or tagged and therefore are unlikely to capture the totality of genetic effects. Given the fundamental differences of what is being measured, we present our results in two main categories: twin-based heritability estimates and SNP-based heritability estimates. We then address details within each category by examining specific methodologies and their impact on the resulting heritability values, providing a comprehensive view of LOAD heritability across diverse study designs and populations.

### Twin-based Heritability

**Fig 2.**
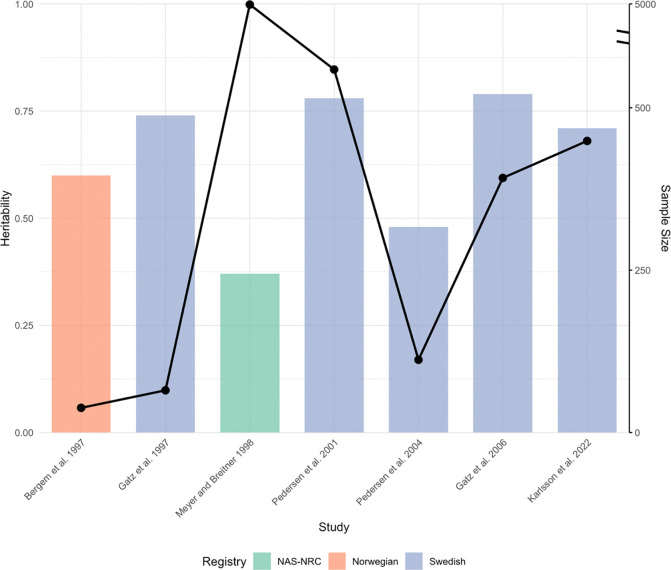
Twin study-based heritability estimates and sample sizes across studies. The figure presents the heritability estimates (bars, left y-axis) and corresponding sample sizes (line, right y-axis) for studies arranged chronologically along the x-axis. Each bar represents a single study, with colors denoting different twin registries utilized. There is a break mark, indicated by a double slash, on the right y-axis between 500 and 4850 to accommodate the wide range of values for sample sizes.

Seven studies using the family-based approach met our inclusion criteria, all of which were based on twin studies. Despite similar study designs and similar estimation approaches, LOAD heritability estimates based on twin studies vary widely, ranging from 37% to 79% (Table 1; Fig 2). We further investigated the potential reasons that could lead to the differences among the estimated values, including variations in twin registries, study population characteristics, and LOAD phenotyping.

### Twin Registries

The majority of the identified studies used the Swedish Twin Registry (STR) [43], with two exceptions: one study leveraged the Norwegian Twin Registry (NTR) [44] and another the National Research Council Registry of Aging Twin Veterans (NAS-NRC), also from Norway [45]. The NTR study had the smallest sample size (n = 38 twin pairs with AD diagnosis) [38], a size expected given the study formulated the target probands via cross-referencing the cognitively impaired elderly born between 1895–1925 within the twin registry. The NAS-NRC panel, established in the mid-1950s, focused on white male twins [46]. The registry’s focus on white males and the high non-participation rate of (24%) of the LOAD study [37] potentially makes its heritability estimates less representative compared with other estimates [45].

The STR-based LOAD heritability studies were more complex than those from the two registries discussed above, involving longitudinal efforts and variability within sub-studies. Three studies leveraged samples from the Study of Dementia in Swedish Twins [39] based on the Swedish Adoption/Twin Study of Aging (SATSA), a subset of the population-based STR with detailed enrollment criteria [47]. These studies included twins drawn from STR with variations in the inclusion criteria across studies, resulting in varying numbers of twin pairs in the analysis. In addition to the SATSA panel, Pedersen et al (2004) also leveraged the Origins of Variance in the Oldest Old: Octogenarian Twins (OCTO-Twin) [48] that incorporated longitudinal observations into the study, resulting in 662 twin pairs without symptoms with follow-ups spanning on average five years [41]. The sample size was further enriched with the introduction of the Study of Dementia in Swedish Twins (HARMONY) initiated in the year 1998 [49]. Prior to the HARMONY study, STR assessments were not comprehensive for dementia, limiting sample sizes. A larger sample size was achieved in the Gatz et al (2006) study (n = 392 pairs with at least 1 AD case) that employed a two-phase phenotype scheme where all participants were initially screened for cognitive dysfunction and suspected AD cases were followed up with complete clinical diagnostic evaluations. This STR LOAD heritability study also included opposite-sex twin pairs [22]. More recent efforts have further expanded the use of STR data by incorporating four STR sub-studies (SATSA, OCTO-Twin, HARMONY, and Aging in Women and Men) [42]. The inclusion of Aging in Women and Men (also known as GENDER) added value by introducing opposite-sex twin pairs to LOAD heritability research [50].

Other twin registries exist that are not included in this review. For example, the Finnish Twin Registry study by Räihä et al. (1996) [51] was also mentioned by Pederson et al (2001) as part of their exploration of LOAD heritability using a single-threshold model, although it was not the primary focus of their research [40]. The original study included same-sex twin pairs from Finland born before the year 1958, with disease status identified through the linkage to the Hospital Discharge Register, leading to a total of 94 AD affected out of 178 twin individuals [51]. Incomplete record linkage could potentially explain the lower heritability estimate of 63% reported by Pedersen et al. (2001) for the Finnish Twin Registry study [40].

### Twin Registry Characteristics

The study populations differed in numerous characteristics that may influence measures of heritability. Notable variation in participants’ ages, a demographic strongly correlated with LOAD prevalence [52], were observed across studies. The twin veterans study had an average age of 63.1 years (SD: 5.4) [37]. This younger sample might not have fully manifested LOAD symptoms at the time of assessment, potentially contributing to a relatively lower heritability estimate (h^2^ = 37%). In contrast, Pedersen et al. (2004) observed a much later average age of 83.9 years (SD: 6.3) [41] and higher heritability (48%; Table 1) that could be attributed to their longitudinal study design, where twin pairs aged 52 to 98 were followed for approximately 5 years. The Karlsson study in 2022, leveraging enriched samples from the STR, benefitted from a longer follow-up (through 2016) resulting in an average age of 85.28 years (SD: 7.0) and higher heritability (71%) compared with both Meyer and Breitner (1988) and Pederson et al (2004) [42]. These age variations highlight the importance of considering age, and when possible, birth cohort, of the participants in LOAD studies to capture a more comprehensive picture of its heritability.

Several studies also explicitly indicated the inclusion of different sex twin pairs. LOAD disproportionally affects females, with higher lifetime risk and sex-specific risk factors observed in previous studies [53,54]. Moreover, prior research indicates that genetic components may manifest differently in males and females [55]. Bergem et al. (1997) was the first study we identified that included twins of differing sex; however, it was limited by a small proportion of male-female twins (33% among the dizygotic twin pairs). Later studies leveraging HARMONY, as described above, achieved a more balanced composition of like- and unlike-sex twin pairs; the former study did not identify differences in heritability across sexes, while the latter also incorporated additional opposite-sex twin pairs drawn from GENDER, representing 21% of the total sample.

One study stands out for its focus on incident LOAD cases rather than prevalent cases [41]. While most studies examine prevalent cases to estimate heritability, this study incorporating two longitudinal twin studies investigated the relative importance of genetic and environmental impact in disease development across different age of onset groups. The unique study design yielded an overall heritability estimate of 48%, with age-stratified estimates of 59% for onset before age 80 and 40% for onset after age 80. These estimates are generally lower than those obtained from studies of prevalent cases (Table 1), revealing the potential impact of study design on heritability estimates.

### Twin Study Modeling Strategies

A straightforward method for heritability estimation is Falconer’s formula [*h*^2^ = 2(*r_MZ_* — *r_DZ_*], which relies on the phenotypic correlation between twin pairs [56]. Bergem et al. (1997) applied Falconer’s formula to estimate the heritability of AD, incorporating a series of probability percentages for positive cases, and obtained heritability estimates ranging from 55% to 61% [38]. An advantage of phenotypic correlation is its ease in interpretability. However, this approach relies heavily on the assumption of equal shared environmental variance and lacks the ability to address more complex aspects of genetic analysis, such as model performance assessment, incorporation of extended family data, and investigation of gene-by-environment interactions, potentially leading to oversimplified estimates that do not fully capture the genetic architecture of complex traits like LOAD.

With advances in analytical techniques and software for effective data handling, more sophisticated modeling approaches have been developed to estimate variance components using information from twin studies. Among these methods, structural equation modeling (SEM) has become frequently employed, with model fitting relying on maximum likelihood estimation. SEM offers greater flexibility in determining the contributions of additive genetic (A), shared environmental (C), and unique environmental (E) components, collectively forming the ACE model, while also allowing for the incorporation of covariates into the analysis, providing a more comprehensive understanding of the factors influencing heritability [11,57]. Typically, these studies involve testing both the full ACE model and reduced models, successively dropping either A or C, to identify the best-fitting model along with the corresponding parameter estimations [22,37,39–42]. Model performance is generally assessed using indicators such as the Akaike Information Criterion (AIC) and chi-square test of difference, while some studies also incorporate critical information, such as consistency with observed correlations and concordance estimates, into the determination of the best-fitting model [39,41].

The majority of the heritability estimates presented in LOAD twin studies (Table 1) originated from the AE model, which excludes shared environmental variance, assuming that additive genetic components and unique environmental impact are the primary contributors to LOAD risk. Exceptions to this approach include Gatz et al. (1997) who reported that the full ACE model performed best, estimating heritability at 74% and attributing 24% to the shared environmental effects [39]. Meyer et al. (1998) found neither the AE nor CE models could be rejected compared to the full model, yielding heritability estimates of 74% in the AE model and 37% in the full model [37]. Collectively, these studies demonstrated the importance of considering multiple models and interpreting results cautiously. The preference for the AE model in the LOAD twin studies does not necessarily negate the relevance of shared environmental factors. Instead, it indicates their contribution may be minor or more difficult to detect given current methodologies, emphasizing the need for larger studies and sophisticated modeling approaches to fully elucidate the complex interplay of factors contributing to disease risks.

In heritability studies of LOAD, analyzing binary traits (affected vs. unaffected) presents unique challenges compared to quantitative traits [58,59]. Dichotomous outcomes necessitate a specialized approach to estimating variance component partitions, typically via the assumption of a latent, normally distributed liability for the trait. Disease status is then determined by a threshold, often corresponding to the population disease prevalence, with individuals above this threshold considered affected, while those below as unaffected. This liability threshold model is necessary for incorporating binary outcomes into a framework suitable for heritability analysis. Thus, it is important to use the appropriate threshold(s) in the study, not only to address the ascertainment bias embedded in the twin study design but also to accurately reflect the population characteristics for age-dependent diseases, like LOAD.

The complexity of this process is evident in the approaches adopted in different studies. Some studies leveraged previously published epidemiological data to inform the liability threshold. Bergem et al. (1997) utilized the published population prevalence of LOAD, setting the prevalence at 10% in their analysis based on previous studies with similar age distributions [60,61]. In contrast, several other studies [22,37,39–41] relied on the estimated prevalence or incidence rates derived from their study samples. While this method potentially offers specificity to the study population, it makes it difficult to compare heritability estimates across studies.

Given the challenges of using prevalence or incidence, some studies focused on a threshold modeling process for LOAD to better capture the potential right-censoring in the data, along with the accurate representation of study samples. This methodology moves beyond the single threshold model used in earlier studies [38,39] to more sophisticated approaches that address the relationship between age of onset and disease liability, such as the implementation of multiple threshold models. Meyer et al. (1998) and Pedersen et al. (2001) incorporated the five-year age groups for monozygotic and dizygotic twin groups, assigning multiple thresholds corresponding to the specific prevalences estimated in these stratified samples [37,40]. Pedersen et al. (2001) further refined this approach by incorporating an additional age group or bin to distinguish censored observations and by testing models with both population-based and sample-specific prevalence estimates [40]. Pedersen et al. (2004) also incorporated differing incidence rates within two age groups (below and above 80 years old) in their heritability analysis. While this approach has the potential to offer a more nuanced understanding of age-related genetic influences on disease onset, no differences were observed in estimates for twins <80 years of age compared with twins ≥80 years of age. [41].

One study not only employed biometrical analysis as discussed above but also incorporated a sex-limitation twin model, leveraging data from a reasonable number of different-sex twin pairs [22]. Unlike previous studies that had a single major stratum based on twin similarity (e.g., monozygotic versus dizygotic), Gatz et al. allowed for estimated thresholds to differ both by sex and age of twins. This more complex modeling offered more insights into variance partitioning, enabling the estimation of sex-specific effects and demonstrating the lack of differences in LOAD heritability between men and women.

### Twin Study Meta-Analyses

In addition to the seven twin-based heritability studies detailed here, two meta-analyses based on family data from individual studies exist in the literature. In one meta-analysis, a weighted mean heritability was estimated based on five previously published twin studies [62]. Among the studies meeting their inclusion criteria, four [22,37–39] are detailed in Table 1 whereas the additional included twin study is from the Finnish twin registry [51]. By weighing the number of twin pairs where at least one is affected by the disorder, the analysis yielded a heritability estimate of 75%. Another meta-analysis of twin correlations from seven studies is available in the literature [63] that includes all of the twin studies described in Table 1 except Karlsson et al. 2022, which was published later. Overall, monozygotic twins for dementia in Alzheimer’s disease had a higher correlation (0.86) compared with dizygotic twins (0.50) (MaTCH) [63]. Using ACE models, the meta-analysis yielded a heritability of 63% for all twins and 59% for the same sex twins [63]. Applying a different method, least squares models based on Falconer’s formula, to the same data led to a higher heritability estimate of 71% for the same-sex twins [63].

### SNP-Based Heritability

Compared with study designs requiring related individuals, population-based approaches for heritability estimation have gained popularity in recent years largely due to the widespread availability of genome-wide SNP data from large case-control studies as well as the advancements in statistical methodologies [36,64–67]. Unlike family-based studies, the population-based approaches utilize the realized genetic matrices computed from the genetic data of large cohorts, circumventing the need for specific family designs and thus broadening the scope for heritability estimation [68]. SNP-based heritability refers to the estimated heritability that is attributable to the assayed or imputed SNPs associated with a complex trait or outcome. Multiple methods have been developed to estimate SNP-based heritability. Some require individual-level data while others require only summary statistics [69].

### GCTA

Genome-wide complex trait analysis (GCTA) is one of the most commonly used methods that leverages individual-level data while relying on different algorithms [70,71]. GCTA employs linear mixed models (LMM) with a normality assumption for the residuals and partitions the phenotypic variance into variance components, leveraging the genetic relatedness matrix (GRM) constructed from the genetic data. The heritability can then be estimated through the genome-based restricted maximum likelihood (GREML) procedure. We identified seven LOAD heritability studies that employed this method (Table 2).

**Fig 3.**
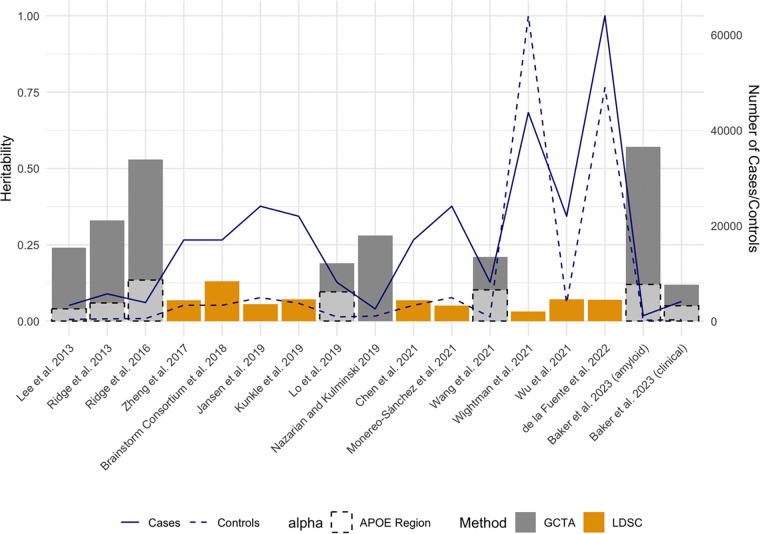
SNP-based heritability and sample sizes across studies. The figure illustrates SNP-based heritability estimates and sample sizes for different studies, ordered by year of publication and author. Bars represent heritability estimates obtained from each study, with different colors indicating the methods used to estimate heritability (GCTA or LDSC). The solid line demonstrates the number of cases in each study, while the dashed line represents the number of controls.

The first LOAD GCTA heritability estimates were published in 2013 (Table 2; Fig 3). The resulting LOAD heritability estimate of 24% was based on a sample of 7,139 participants (3,290 cases, 3,849 controls) that included both elderly screened and population controls [72,73]. Concurrently, Ridge et al. (2013) leveraged the Alzheimer’s Disease Genetics Consortium (ADGC) dataset [74], including 10,922 individuals (5,708 cases, 5,214 controls), and estimated a higher heritability estimate of 33% [75]. The ADGC dataset has since continued to expand, facilitating more comprehensive analyses of LOAD. A 2016 update included data from 30 studies within the ADGC, and the heritability estimate increased to 53%. This study included only 9,699 individuals (3,877 cases, 5,822 controls) [76]. This sample size was smaller in this latter study due to the requirement for non-missing data across 21 known AD genes, resulting in a higher LOAD heritability estimate compared with the 2013 analysis and highlighting the impact of the quality control (QC) process and demand for complete data at known LOAD risk loci. Inclusion of more known LOAD genes increased the heritability, indicating that these genes have substantial impact on overall estimates.

Later and larger two-phase ADGC datasets focusing on age- and sex-specific heritability utilized samples of up to 17,896 participants [77,79]. Unlike the smaller twin study of Pederson et al (2004) that found no difference in LOAD heritability by age, Lo et al (2019) with a much larger sample size of unrelated individuals estimated a higher heritability for participants >80 years of age (n=5,198; h^2^=24.1%) compared with those aged 60–69 years of age (n=12,698; h^2^=16.9%). Differences by sex were subtler with a slightly higher LOAD heritability among women (21.5%) compared with men (19.5%) [79]. Overall, variation in study design, case definition, and sample characteristics can greatly impact heritability estimates, necessitating a more cautious interpretation of these findings.

The evolution of LOAD genetic studies has been marked not only by increasing sample sizes but also by a substantial enhancement in marker density. This improvement can be attributed to advancements in both genotyping technologies and imputation techniques, significantly impacting the comprehensiveness of SNP-based heritability estimates. The relatively lower heritability estimation (h^2^ = 24%) obtained by Lee et al (2013) can be partially attributed to the less dense genotype data, utilizing only 499,757 SNPs [73]. This limitation in marker density likely constrained its ability to fully capture SNP-based heritability, which was also supported by the observed decrease in heritability estimates when more stringent QC processes were applied. As opposed to being solely dependent on the directly genotyped data, subsequent studies have leveraged the power of imputation, hugely increasing the number of genetic variants analyzed. In the same year, Ridge et al. (2013) exemplified this leap forward by employing data imputed against the HapMap phase II (release 22) reference panel [81], resulting in 2,042,116 SNPs after quality control, a substantial increase compared to non-imputed datasets that all had fewer than 500,000 SNPs [75]. The trend towards higher marker density has been further accelerated by advances in imputation reference panels. The adoption of the 1000 Genomes Project reference panel [82], offering more comprehensive coverage, has enabled even denser imputation. Ridge et al. (2016) utilized 8,712,879 SNPs in their analysis, while for more recent studies leveraging the two-phase ADGC data, up to 38 million SNPs were incorporated in the analysis [77,79]. The substantial increase in marker density, from ~500,000 to 38 million SNPs, represents a significant methodological advancement in LOAD genetic studies albeit with new analytical challenges, including more stringent requirements for QC processes and increased computational demands. While the differences in marker density could potentially explain some of the variations in heritability estimates across studies, they alone do not explain the observation that the less dense dataset of Ridge et al (2016) has a much higher heritability (53%) compared with either Lo et al (19%; 2019) or Wang et al (21%; 2021).

Analyses with fewer samples and SNPs provided higher estimates, but the analyses differed in ways that may inform our understanding of heritability estimates. For example, inclusion of specific covariates and variable assumed prevalences, among other variables such as marker density (**Error! Reference source not found.**), complicate comparisons across studies and heritability expectations. As noted above, the GCTA approach that uses LMM allows for the inclusion of covariates as fixed effects, enabling more accurate heritability estimates for LOAD. Age and sex, well-established factors affecting LOAD risk, have been consistently included as covariates in most studies, with the notable exception of Lee et al. (2013) [73]. Principal components (PCs) are universally recognized as crucial for adjusting population structure in genetic studies [83]; however, there is considerable variation in both the number of PCs included in the analyses and how the PCs themselves are calculated. While most studies incorporate 10 PCs [73,75–77,79], Nazarian and Kulminski (2019) used only the first five PCs (Nazarian and Kulminski 2019), and Baker et al (2023) employed a flexible approach where the number of PCs was cohort specific [80]. PCs can be calculated within individual cohorts, as demonstrated by Lo et al. (2019), or using the entire combined dataset as did Ridge et al [76]. These methodological heterogeneities may also contribute to differences observed across heritability estimates.

Methodological heterogeneity when using large collaborative studies to estimate SNP-based heritability with GCTA is not limited to PCs. In practice, large collaborative studies are made up of different cohorts that have undergone varied ascertainment processes and genotyping strategies. While increased sample size and diversity are valuable, they come at the cost of potential heterogeneity across cohorts. Lo et al. (2019) made a significant methodological advance by incorporating cohort indicators into their heritability estimation [77]. This approach yielded a lower estimate of 19% for LOAD compared to previous studies. Their sensitivity analysis showed an increase to 32% when cohort indicators were removed, revealing a substantial cohort effect. Wang et al. (2021) similarly incorporated cohort indicators in their analysis of 2-phase ADGC data, obtaining an expected comparable heritability estimate of 21% [79]. While GCTA-based approaches have significantly advanced our understanding of LOAD heritability, they underscore the need for cautious interpretation of estimates due to methodological variation, particularly in covariate handling, highlighting the importance of continued refinement of analytical strategies. We also cannot ignore the possibility that cohorts within a single study actually have variation in true heritability due to unmeasured environmental parameters.

LOAD phenotyping approaches across studies used to estimate heritability are highly heterogeneous. The majority of heritability estimates, particularly those utilizing data from ADGC, rely on a combination of clinical diagnosis, histopathologic findings, and in some cases, biomarkers. Clinical diagnosis have inherent misdiagnosis rates [84] that can affect heritability estimates. Baker et al (2023) illustrated this by demonstrating that the histopathologically confirmed LOAD yielded higher heritability estimates (31% - 57%) compared to clinically diagnosed LOAD (12% - 32%) when applying a consistent model with a 5% liability threshold to five independent cohorts [80]. Notably, within the Amsterdam Dementia Cohort, using the amyloid-confirmed cases showed a heritability estimate of 57%, while the clinical diagnosed cases from the same population yielded an estimate of 25%. This marked difference emphasizes the significant impact of diagnostic criteria on heritability estimation, potentially helping explain some of the observed variability in heritability across studies.

SNP-based heritability estimates of LOAD must address *APOE*, the major genetic risk factor for LOAD [23]. Compared with other complex trait-associated genetic variation, *APOE* has an outsized effect and alone contributes substantially to LOAD heritability, accounting for an estimated 4% to 13.42% of the total phenotypic variance (Fig 3) [73,75–77,79]. The lowest heritability estimate (4%) was obtained using proxy SNPs for *APOE* [73], and although not significantly different from the proxy-based estimate, two higher heritability estimates for *APOE* were obtained when the *APOE* ε2 and ε4 alleles were directly genotyped [75,76]. In contrast to the SNP partitioning method, some studies employed an approach leveraging the best linear unbiased prediction (BLUP) and involved regressing on the number of *APOE* ε4 alleles to estimate heritability attributable specifically to the *APOE* ε4 alleles [77,79]. Regardless of the variability, published estimates confirm that *APOE* explains a substantial portion of the genetic risk for LOAD.

### LDSC

With the proliferation of large-scale GWAS, and their accompanying summary statistics, methods to estimate heritability such as linkage disequilibrium (LD) score regression (LDSC) have become popular. LDSC only requires summary-level data and involves the process of LD score calculations using the appropriate reference panel for each variant. These calculations can then be incorporated into a regression model where the observed GWAS summary statistics are regressed against the pre-computed LD scores to compute the heritability estimation [85,86]. While convenient and in some cases the only option for difficult-to-access datasets, such methods typically lead to an underestimation of SNP-based heritability compared with those derived from individual-level data [36,87] as evident for LOAD (Tables 2 and 3). We identified nine LOAD heritability studies that employed LDSC (Table 3).

Four major GWAS of LOAD have been pivotal in heritability estimation using LDSC (Table 3; Fig 3), all involving direct phenotyping of cases [24–26,91]. As discussed earlier, multi-consortia collaborative efforts revolutionized large-scale genetic studies of LOAD but also introduced heterogeneity into both study design and included populations, leading to variability in heritability estimates. Beginning with the LOAD GWAS published by Lambert et al. (2013), its stage 1 summary statistics formed the basis for three subsequent LOAD heritability estimates [88–90]. This foundational study involved a meta-analysis of four consortia (the ADGC, the Cohorts for Heart and Aging Research in Genomic Epidemiology (CHARGE) Consortium, the European Alzheimer’s Disease Initiative (EADI), and the Genetic and Environmental Risk in Alzheimer’s Disease (GERAD) Consortium) under the International Genomics of Alzheimer’s Project (IGAP), with a total of 54,162 participants in the analysis. Building on these efforts, Kunkle et al. (2019) leveraged the expanded IGAP study to become one of the largest GWAS of LOAD to date [25]. This comprehensive study incorporated 46 cohorts from the IGAP, including 17 new ones, increasing the sample to 63,926 individuals. Two LOAD heritability estimates were based solely on the stage 1 discovery sample [25,93]. Jansen et al. (2019) performed GWAS utilizing not only IGAP data but also incorporating two additional independent consortia: the Psychiatric Genomics Consortium (PGC-ALZ) and the Alzheimer’s Disease Sequencing Project (ADSP), boosting the total sample size to 79,145 [91,92]. Wightman et al. (2021) further expanded the scope of LOAD genetic research by including additional cohorts from Europe and the US not previously considered in Jansen et al. (2019), resulting in a sample size of 761,702 (43,725 cases, 717,979 controls) and its own heritability estimate based on this LOAD GWAS [26]. All of these large GWAS included cases and controls directly phenotyped for LOAD status.

In contrast to the direct LOAD phenotyping approaches described above, Marioni et al. (2018) contributed a study using proxy-phenotypes based on family history (GWAX), leveraging data from large electronic health records available for UK Biobank participants ages 40 to 69 [95]. Using an innovative approach, de la Fuente et al. (2022) broadened the heritability estimation of LOAD by integrating both GWAS and GWAX while addressing the attenuated heritability estimates when directly combining the summary statistics from the two [94].

Beyond the heterogeneity across cohorts, variations in heritability estimates are observed even within studies using the same LOAD summary statistics. This variability could stem from the unique challenges in estimating heritability for binary traits like LOAD, as compared to quantitative traits. The process of estimating heritability for binary traits involves an initial estimation made on the observed scale, using the sample prevalence as the threshold followed by a transformation to the liability scale utilizing the population prevalence [96,97]. This approach results in a mix of observed- and liability-scale heritability estimates across studies, even when leveraging the same summary statistics, contributing significantly to the diverse heritability values reported in the literature. The impact of this methodological variation on the heritability estimates can be seen in studies leveraging summary statistics [24]. Zheng et al. (2017) and Chen et al. (2021) reported similar observed-scale heritability estimates of 6.88% and 6.80%, respectively [88,90]. In contrast, the Brainstorm Consortium et al. (2018) provided a markedly different liability-scale heritability estimate of 13%, using a population prevalence of 17% for the transformation [89]. Liability scale heritability is critical in adjusting for the ascertainment of the binary traits and making the heritability estimates comparable across studies using appropriate population prevalence for transformation. The challenge of interpreting and comparing these mixed-scale estimates is further compounded by the wide range of population prevalence figures used in liability-scale transformations. Across various studies, these prevalence estimates range from 4.3% to 17% [26,89,91,94]. This variability in prevalence estimates can significantly impact heritability figures, making direct comparisons between studies difficult.

### SNP-Based Heritability Re-Evaluation

In addition to the 16 SNP-based studies (Tables 2 and 3) included in this review that met our criteria (Fig 1), we identified several “re-evaluations” of LOAD SNP-based heritability where the heritability estimates were primarily generated in the context of gene discoveries. With variation in the data included, the methodology considered, and assumptions made, these re-evaluations based on previous studies resulted in a range of incomparable estimates. At least three SNP-based heritability re-evaluations studies have been published: one utilizing both GCTA and LDSC [98], one using only LDSC [99], and a third one employing LDAK [100], a method that accounts for minor allele frequency and LD when estimating a SNP’s influence on a trait’s heritability [101,102]. The number of datasets or studies included ranged from four [98] to ten [99] that resulted in a large range of heritability estimates with a consistent population prevalence of 5%: the LOAD heritability estimates using LDSC ranged from 9% to 17% [98] and 3% to 42% [99]. As expected, the GCTA estimates were higher at 25% to 31% [98]. LDAK estimates generally aligned more closely with GCTA, and estimates derived from LDAK were generally higher than LDSC. As an example, heritability estimates using data from Kunkle et al (2019) increased from 7% with LDSC (Table 3) to 21% using LDAK [100].

## Conclusion and Discussion

**Fig 4.**
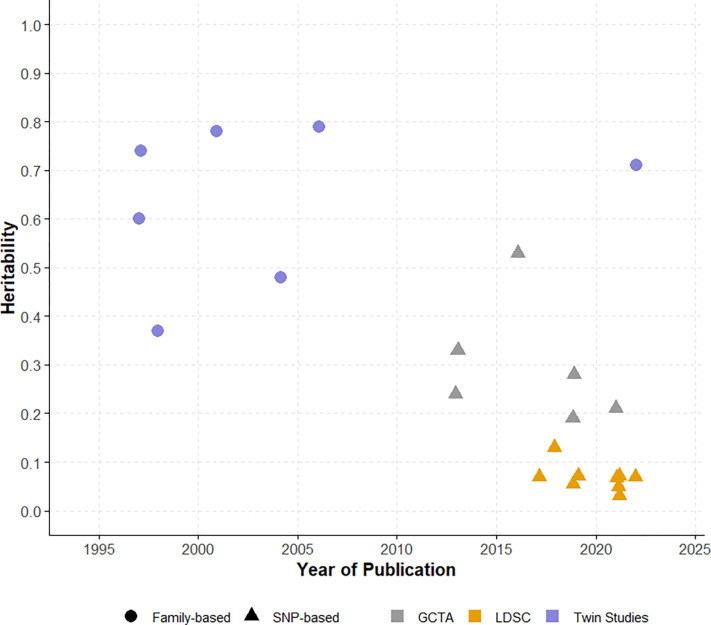
Heritability estimates across studies over time. Here shows the heritability estimates for LOAD from all studies identified, published between 1997 and 2022. Within the figure, each data point represents an individual estimate corresponding to the value on the y-axis, with shapes distinguishing between family-based (circle) and SNP-based (triangle) approaches, while colors indicating specific methods: purple for twin studies, gray for GCTA, and orange for LDSC.

In this review we evaluated multiple measures of heritability for LOAD and showed that direct comparisons between heritability studies are difficult due to study population variability, methodological differences, and other factors outlined in this review. Understanding and critically evaluating heritability estimates require careful consideration of underlying assumptions, methodological nuances, and the challenges inherent in quantifying genetic contributions to complex diseases like LOAD. Another significant limitation of current LOAD heritability research is its predominant focus on European descent populations. This lack of diversity, well-documented in genetic studies [103], substantially impacts the generalizability and broader applicability of obtained estimates. However, it is of note that all of the studies we reviewed were of European ancestry and variation in heritability estimates was still huge. Moving forward, the recognition that there is no one real measure of heritability should be promoted, reinforcing that each estimate captures nested components of genetic architecture within the specific methodological and population context from which they were obtained. Clearly, future studies would benefit from increased population diversity, standardized phenotyping approaches, and careful consideration of both genetic and environmental factors to provide a more comprehensive understanding of LOAD heritability across diverse populations. Despite the limitations in estimating LOAD heritability, doing so is still important as LOAD is a neurodegenerative disorder with significant public health implications, and knowledge of heritability is a gateway to better understanding its genetic architecture and guiding future research efforts [3,67,68,104]. In this systematic review, we identified 23 studies providing LOAD heritability estimates, employing either twin-based (n=7) or SNP-based (n=16) approaches. The included studies demonstrated a range of heritability estimates that varied considerably (Fig 4), reflecting the diversity in study designs, estimation methodologies, and the underlying heterogeneity of LOAD. Family-based approaches that leverage the genetic similarities between relatives to estimate heritability, generally yielded higher estimates, ranging from 37% to 89% based on twin studies. The higher estimates for twin studies are expected as these implicitly include all genetic variants and their possible non-additive effects, while also potentially including shared environmental effects that are difficult to dissect. This potentially leads to an overestimation of heritability. In contrast, SNP-based approaches that compute heritability estimates based on measured genetic variants across the genome, provided relatively lower estimates (3.1% to 53%), as they are limited by what has been genotyped or imputed [59]. Among them, studies using summary statistics provided relatively lower estimates (LDSC-based; 3.1% to 13%) compared with those using genotyped or imputed data (GCTA-based; 19% to 53%). As we recognize that LDSC-based heritability estimates tend to be downwardly biased [87,102,105,106], in contrast to twin studies that can capture gene-environment interactions, along with other factors, that potentially inflate heritability estimates [107–109]. While acknowledging that the heritability of LOAD is variable to some extent [80], we posit that the heritability of LOAD in most European-descent populations falls between 40–60%, capturing the upper range of SNP-based heritability estimates and the lower range of twin-based estimates. We think that this range better represents the substance of LOAD heritability than any single point estimate from any given study.

## Figures and Tables

**Table 1.** Twin Study-based LOAD heritability studies.

Study	Phenotype/Twin Registry (Population)	h^2^	MZ Age of Onset (SD)	DZ Age of Onset (SD)	Sample Size (# twin pairs with at least 1 case)	% Female	Opposite-Sex Twin Pairs

Bergem et al. 1997 [38]	Norwegian (EUR)	60%	79 (6.6)		38	78%	Yes
Gate et al. 1997 [39]	Swedish (EUR)	74%	75.8 (9.03)	73.7 (8.92)	65	ND	No
Meyer and Breitner 1998 [37]	NAS-NRC (EUR)	37%*	63.1 (5.4)		4897 ^(1)^	0%	No
Pedersen et al. 2001 [40]	Swedish (EUR)	78%^	75		559 ^(2)^	ND	No
Pedersen et al. 2004 [41]	Swedish^+^ (EUR)	48%^	83.9 (6.3)		112	ND	No
Gate et al. 2006 [22]	Swedish (EUR)	79%^	78.1		392	56%	Yes
Karlsson et al. 2022 [42]	Swedish (EUR)	71%^	85.28 (7.02)		449	56%	Yes

+ Incident cases

^ Values presented are findings from the reduced AE model, where the C parameter (shared environmental component) was dropped, while the remaining heritability estimates were obtained from the full ACE model.

* Full ACE model result shown, while the original study indicated that neither reduced AE nor CE model could be statistically rejected due to limited AD cases.

(1) Total of complete twin pairs, with 39 cases

(2) Total of twin pairs for longitudinal observation

Abbreviations: ND: no data, MZ: monozygotic twins, DZ: dizygotic twins, SD: standard deviation, NAS-NRC: National Research Council Registry of Aging Twin Veterans, EUR: European descent or of European ancestry

**Table 2.** SNP-based LOAD heritability studies using GCTA-GREML.

Study (Population)	h^2^	h^2^ SE	Sample Size	Age of Onset: Cases	Age of Onset: Controls	# SNPs	Covariates	Prevalence for liability transformation

Lee et al. 2013 [73] (EUR)	24%	3%	7,139	ND		499,757^	10 PCs	2.00%
Ridge et al. 2013 [75] (EUR)	33%	3%	10,922	74.3	75.9	2,042,116	Age, sex, and 10 PCs	13.00%
Ridge et al. 2016 [76] (EUR)	53%	4%	9,699	77.7		8,712,879	Age, sex, and 10 PCs	13.00%
Lo et al. 2019 [77] (EUR)	19%	2%	17,896	ND		38,043,082	Age, sex, cohort indicators, and 10 PCs	6.13%
Nazarian and Kulminski 2019 [78] (EUR)	28%*	9%	14,283	84.3	69.6	~1.5–2 million	Age, sex, and 5 PCs	10.00%
Wang et al. 2021 [79] (EUR)	21%	2%	17,896	ND		38,043,082	Age, cohort indicators, and 10 PCs	5.50% in males 7.20% in females
Baker et al. 2023^+^ [80] (EUR)	12%–57%	-	803 – 12,528	77.0	69.6	ND	Age, sex and 5–15 PCs§	5.00%

* Estimated from three independent datasets via an inverse-variance meta-analysis

^ Without imputation

+ Included estimates from five different independent cohorts. The heritability and sample size presented represent range across these cohorts (SE for heritability not shown), while age of onset reflect the average across the whole sample.

§ Number of PCs is dependent on the cohort

Abbreviations: ND: not data, SE: standard error, PC: principal components, EUR: European descent or of European ancestry

**Table 3.** SNP-based LOAD heritability studies using LDSC.

Study	h^2^	h^2^ SE	Prevalence for liability transformation	Summary Statistics (Population)	Sample Size

Zheng et al. 2017 [88]	6.88%	1.30%	ND		
Brainstorm Consortium et al. 2018 [89]	13%^	2.30%	17.00%	Lambert et al. 2013 [24] (EUR)	54,162
Chen et al. 2021 [90]	6.80%	ND	ND		

Jansen et al. 2019 [91]	5.50%	1%	4.30%	Jansen et al. 2019 [91] (EUR)	79,145
Monereo-Sánchez et al. 2021 [92]	5%^	1%	ND		

Kunkle et al. 2019 [25]	7.10%	1.10%	ND	Kunkle et al. 2019 [25] (EUR)	63,926
Wu et al. 2021 [93]	7.13%	1.14%	ND		

Wightman et al. 2021 [26]	3.10%	0.60%	5.00%	Wightman et al. 2021 [26] (EUR)	761,704

de la Fuente et al. 2022 [94]	6.90%^	0.80%	5.00%	Kunkle et al. 2019 [25] & Marioni et al. 2018* [95] (EUR)	612,881

* Study included proxy cases

^ The region surrounding the *APOE* locus removed from LD scores calculation

Abbreviations: ND: no data, SE: standard error, EUR: European descent or of European ancestry

## References

[1] Lynch M, Walsh B. Genetics and analysis of quantitative traits. invemar.org.co; 1998 [cited 9 Jan 2024]. Available: https://www.invemar.org.co/redcostera1/invemar/docs/RinconLiterario/2011/febrero/AG_8.pdf

[2] Fisher RA. XV.—The Correlation between Relatives on the Supposition of Mendelian Inheritance. Earth Environ Sci Trans R Soc Edinb. 1919;52: 399–433.

[3] Visscher PM, Hill WG, Wray NR. Heritability in the genomics era — concepts and misconceptions. Nat Rev Genet. 2008;9: 255–266. 18319743 10.1038/nrg2322

[4] Robette N, Génin E, Clerget-Darpoux F. Heritability: What’s the point? What is it not for? A human genetics perspective. Genetica. 2022;150: 199–208. 35092541 10.1007/s10709-022-00149-7

[5] Silventoinen K, Kaprio J, Lahelma E, Koskenvuo M. Relative effect of genetic and environmental factors on body height: differences across birth cohorts among Finnish men and women. Am J Public Health. 2000;90: 627–630. 10754982 10.2105/ajph.90.4.627 PMC1446206

[6] Perola M, Ohman M, Hiekkalinna T, Leppävuori J, Pajukanta P, Wessman M, Quantitative-trait-locus analysis of body-mass index and of stature, by combined analysis of genome scans of five Finnish study groups. Am J Hum Genet. 2001;69: 117–123. 11410840 10.1086/321286 PMC1226026

[7] Maher B. Personal genomes: The case of the missing heritability. Nature. 2008;456: 18–21. 18987709 10.1038/456018a

[8] Manolio TA, Collins FS, Cox NJ, Goldstein DB, Hindorff LA, Hunter DJ, Finding the missing heritability of complex diseases. Nature. 2009;461: 747–753. 19812666 10.1038/nature08494 PMC2831613

[9] Yengo L, Vedantam S, Marouli E, Sidorenko J, Bartell E, Sakaue S, A saturated map of common genetic variants associated with human height. Nature. 2022;610: 704–712. 36224396 10.1038/s41586-022-05275-y PMC9605867

[10] Wainschtein P, Jain D, Zheng Z, TOPMed Anthropometry Working Group, NHLBI Trans-Omics for Precision Medicine (TOPMed) Consortium, Cupples LA, Assessing the contribution of rare variants to complex trait heritability from whole-genome sequence data. Nat Genet. 2022;54: 263–273. 35256806 10.1038/s41588-021-00997-7 PMC9119698

[11] Tenesa A, Haley CS. The heritability of human disease: estimation, uses and abuses. Nat Rev Genet. 2013;14: 139–149. 23329114 10.1038/nrg3377

[12] Srivastava AK, Williams SM, Zhang G. Heritability estimation approaches utilizing genome-wide data. Curr Protoc. 2023;3: e734. 37068172 10.1002/cpz1.734 PMC10923601

[13] Friedman NP, Banich MT, Keller MC. Twin studies to GWAS: there and back again. Trends Cogn Sci. 2021;25: 855–869. 34312064 10.1016/j.tics.2021.06.007 PMC8446317

[14] Hoffmann AA, Merilä J. Heritable variation and evolution under favourable and unfavourable conditions. Trends Ecol Evol. 1999;14: 96–101. 10322508 10.1016/s0169-5347(99)01595-5

[15] Charmantier A, Garant D. Environmental quality and evolutionary potential: lessons from wild populations. Proc Biol Sci. 2005;272: 1415–1425. 16011915 10.1098/rspb.2005.3117 PMC1559820

[16] Kruuk LEB, Hadfield JD. How to separate genetic and environmental causes of similarity between relatives. J Evol Biol. 2007;20: 1890–1903. 17714306 10.1111/j.1420-9101.2007.01377.x

[17] Wilson AJ, Réale D, Clements MN, Morrissey MM, Postma E, Walling CA, An ecologist’s guide to the animal model. J Anim Ecol. 2010;79: 13–26. 20409158 10.1111/j.1365-2656.2009.01639.x

[18] Silventoinen K, Sammalisto S, Perola M, Boomsma DI, Cornes BK, Davis C, Heritability of adult body height: A comparative study of twin cohorts in eight countries. Twin Res. 2003;6: 399–408. 14624724 10.1375/136905203770326402

[19] Yang J, Bakshi A, Zhu Z, Hemani G, Vinkhuyzen AAE, Lee SH, Genetic variance estimation with imputed variants finds negligible missing heritability for human height and body mass index. Nat Genet. 2015;47: 1114–1120. 26323059 10.1038/ng.3390 PMC4589513

[20] Reitz C, Brayne C, Mayeux R. Epidemiology of Alzheimer disease. Nat Rev Neurol. 2011;7: 137–152. 21304480 10.1038/nrneurol.2011.2 PMC3339565

[21] 2024 Alzheimer’s disease facts and figures. Alzheimers Dement. 2024;20: 3708–3821. 38689398 10.1002/alz.13809 PMC11095490

[22] Gatz M, Reynolds CA, Fratiglioni L, Johansson B, Mortimer JA, Berg S, Role of genes and environments for explaining Alzheimer disease. Arch Gen Psychiatry. 2006;63: 168–174. 16461860 10.1001/archpsyc.63.2.168

[23] Corder EH, Saunders AM, Strittmatter WJ, Schmechel DE, Gaskell PC, Small GW, Gene dose of apolipoprotein E type 4 allele and the risk of Alzheimer’s disease in late onset families. Science. 1993;261: 921–923. 8346443 10.1126/science.8346443

[24] Lambert JC, Ibrahim-Verbaas CA, Harold D, Naj AC, Sims R, Bellenguez C, Meta-analysis of 74,046 individuals identifies 11 new susceptibility loci for Alzheimer’s disease. Nat Genet. 2013;45: 1452–1458. 24162737 10.1038/ng.2802 PMC3896259

[25] Kunkle BW, Grenier-Boley B, Sims R, Bis JC, Damotte V, Naj AC, Genetic meta-analysis of diagnosed Alzheimer’s disease identifies new risk loci and implicates Aβ, tau, immunity and lipid processing. Nat Genet. 2019;51: 414–430. 30820047 10.1038/s41588-019-0358-2 PMC6463297

[26] Wightman DP, Jansen IE, Savage JE, Shadrin AA, Bahrami S, Holland D, A genome-wide association study with 1,126,563 individuals identifies new risk loci for Alzheimer’s disease. Nat Genet. 2021;53: 1276–1282. 34493870 10.1038/s41588-021-00921-z PMC10243600

[27] Bellenguez C, Küçükali F, Jansen IE, Kleineidam L, Moreno-Grau S, Amin N, New insights into the genetic etiology of Alzheimer’s disease and related dementias. Nat Genet. 2022;54: 412–436. 35379992 10.1038/s41588-022-01024-z PMC9005347

[28] Falconer DS, Mackay FCT. Introdutction to Quantitative Genetics. Introdutction to Quantitative Genetics. 1996. pp. 464–464.

[29] Ossom Williamson P, Minter CIJ. Exploring PubMed as a reliable resource for scholarly communications services. J Med Libr Assoc. 2019;107: 16–29. 30598645 10.5195/jmla.2019.433 PMC6300231

[30] Plassman BL, Breitner JC. The genetics of dementia in late life. Psychiatr Clin North Am. 1997;20: 59–76. 9139296 10.1016/s0193-953x(05)70393-2

[31] Wingo TS, Lah JJ, Levey AI, Cutler DJ. Autosomal recessive causes likely in early-onset Alzheimer disease. Arch Neurol. 2012;69: 59–64. 21911656 10.1001/archneurol.2011.221 PMC3332307

[32] Page MJ, McKenzie JE, Bossuyt PM, Boutron I, Hoffmann TC, Mulrow CD, The PRISMA 2020 statement: an updated guideline for reporting systematic reviews. Syst Rev. 2021;10: 89. 33781348 10.1186/s13643-021-01626-4 PMC8008539

[33] Sabatti C, Service SK, Hartikainen A-L, Pouta A, Ripatti S, Brodsky J, Genome-wide association analysis of metabolic traits in a birth cohort from a founder population. Nat Genet. 2009;41: 35–46. 19060910 10.1038/ng.271 PMC2687077

[34] Speed D, Hemani G, Johnson MR, Balding DJ. Improved heritability estimation from genome-wide SNPs. Am J Hum Genet. 2012;91: 1011–1021. 23217325 10.1016/j.ajhg.2012.10.010 PMC3516604

[35] Gusev A, Bhatia G, Zaitlen N, Vilhjalmsson BJ, Diogo D, Stahl EA, Quantifying missing heritability at known GWAS loci. PLoS Genet. 2013;9: e1003993. 24385918 10.1371/journal.pgen.1003993 PMC3873246

[36] Yang J, Zeng J, Goddard ME, Wray NR, Visscher PM. Concepts, estimation and interpretation of SNP-based heritability. Nat Genet. 2017;49: 1304–1310. 28854176 10.1038/ng.3941

[37] Meyer JM, Breitner JC. Multiple threshold model for the onset of Alzheimer’s disease in the NAS-NRC twin panel. Am J Med Genet. 1998;81: 92–97. 9514594 10.1002/(sici)1096-8628(19980207)81:1<92::aid-ajmg16>3.0.co;2-r

[38] Bergem AL, Engedal K, Kringlen E. The role of heredity in late-onset Alzheimer disease and vascular dementia. A twin study. Arch Gen Psychiatry. 1997;54: 264–270. 9075467 10.1001/archpsyc.1997.01830150090013

[39] Gatz M, Pedersen NL, Berg S, Johansson B, Johansson K, Mortimer JA, Heritability for Alzheimer’s disease: the study of dementia in Swedish twins. J Gerontol A Biol Sci Med Sci. 1997;52: M117–25. 9060980 10.1093/gerona/52a.2.m117

[40] Pedersen NL, Posner SF, Gatz M. Multiple-threshold models for genetic influences on age of onset for Alzheimer disease: findings in Swedish twins. Am J Med Genet. 2001;105: 724–728. 11803520 10.1002/ajmg.1608

[41] Pedersen NL, Gatz M, Berg S, Johansson B. How heritable is Alzheimer’s disease late in life? Findings from Swedish twins. Ann Neurol. 2004;55: 180–185. 14755721 10.1002/ana.10999

[42] Karlsson IK, Escott-Price V, Gatz M, Hardy J, Pedersen NL, Shoai M, Measuring heritable contributions to Alzheimer’s disease: polygenic risk score analysis with twins. Brain Commun. 2022;4: fcab308. 35169705 10.1093/braincomms/fcab308 PMC8833403

[43] Cederlöf R, Lorich U. The Swedish Twin Registry. Prog Clin Biol Res. 1978;24 Pt B: 189–195. 569308

[44] Kringlen E. Norwegian twin registers. Prog Clin Biol Res. 1978;24 Pt B: 185–187. 569307

[45] Breitner JC, Welsh KA, Gau BA, McDonald WM, Steffens DC, Saunders AM, Alzheimer’s disease in the National Academy of Sciences-National Research Council Registry of Aging Twin Veterans. III. Detection of cases, longitudinal results, and observations on twin concordance. Arch Neurol. 1995;52: 763–771. 7639628 10.1001/archneur.1995.00540320035011

[46] Page WF. The NAS-NRC Twin Registry of WWII military veteran twins. National Academy of Sciences-National Research Council. Twin Res. 2002;5: 493–496. 12537883 10.1375/136905202320906345

[47] Pedersen NL, McClearn GE, Plomin R, Nesselroade JR, Berg S, DeFaire U. The Swedish Adoption Twin Study of Aging: an update. Acta Genet Med Gemellol. 1991;40: 7–20. 1950353 10.1017/s0001566000006681

[48] McClearn GE, Johansson B, Berg S, Pedersen NL, Ahern F, Petrill SA, Substantial genetic influence on cognitive abilities in twins 80 or more years old. Science. 1997;276: 1560–1563. 9171059 10.1126/science.276.5318.1560

[49] Gatz M, Fratiglioni L, Johansson B, Berg S, Mortimer JA, Reynolds CA, Complete ascertainment of dementia in the Swedish Twin Registry: the HARMONY study. Neurobiol Aging. 2005;26: 439–447. 15653172 10.1016/j.neurobiolaging.2004.04.004

[50] Gold CH, Malmberg B, McClearn GE, Pedersen NL, Berg S. Gender and health: a study of older unlike-sex twins. J Gerontol B Psychol Sci Soc Sci. 2002;57: S168–76. 11983743 10.1093/geronb/57.3.s168

[51] Räihä I, Rajala T, Sourander L, Kaprio J, Koskenvuo M. Alzheimer’s disease in Finnish twins. Lancet. 1996;347: 573–578. 8596319 10.1016/s0140-6736(96)91272-6

[52] Rajan KB, Weuve J, Barnes LL, McAninch EA, Wilson RS, Evans DA. Population estimate of people with clinical Alzheimer’s disease and mild cognitive impairment in the United States (2020–2060). Alzheimers Dement. 2021;17: 1966–1975. 34043283 10.1002/alz.12362 PMC9013315

[53] Plassman BL, Langa KM, Fisher GG, Heeringa SG, Weir DR, Ofstedal MB, Prevalence of dementia in the United States: the aging, demographics, and memory study. Neuroepidemiology. 2007;29: 125–132. 17975326 10.1159/000109998 PMC2705925

[54] Mielke MM. Sex and Gender Differences in Alzheimer’s Disease Dementia. Psychiatr Times. 2018;35: 14–17. 30820070 PMC6390276

[55] Farrer L, Cupples L, Haines J, Hyman B, Kukull W, Mayeux R, Effects of age, sex, and ethnicity on the association between apolipoprotein E genotype and Alzheimer disease: A meta-analysis. JAMA. 1997;278: 1349–1356. 9343467

[56] Falconer DS, MacKay TFC. Introduction to Quantitative Genetics. 4th ed. London, England: Longman; 1995.

[57] Rijsdijk FV, Sham PC. Analytic approaches to twin data using structural equation models. Brief Bioinform. 2002;3: 119–133. 12139432 10.1093/bib/3.2.119

[58] Falconer DS. The inheritance of liability to certain diseases, estimated from the incidence among relatives. Ann Hum Genet. 1965;29: 51–76.

[59] Witte JS, Visscher PM, Wray NR. The contribution of genetic variants to disease depends on the ruler. Nat Rev Genet. 2014;15: 765–776. 25223781 10.1038/nrg3786 PMC4412738

[60] Rocca WA, Hofman A, Brayne C, Breteler MM, Clarke M, Copeland JR, Frequency and distribution of Alzheimer’s disease in Europe: a collaborative study of 1980–1990 prevalence findings. The EURODEM-Prevalence Research Group. Ann Neurol. 1991;30: 381–390. 1952826 10.1002/ana.410300310

[61] Engedal K, Haugen PK. The prevalence of dementia in a sample of elderly norwegians. Int J Geriatr Psychiatry. 1993;8: 565–570.

[62] Bienvenu OJ, Davydow DS, Kendler KS. Psychiatric “diseases” versus behavioral disorders and degree of genetic influence. Psychol Med. 2011;41: 33–40. 20459884 10.1017/S003329171000084X PMC10885735

[63] Polderman TJC, Benyamin B, de Leeuw CA, Sullivan PF, van Bochoven A, Visscher PM, Meta-analysis of the heritability of human traits based on fifty years of twin studies. Nat Genet. 2015;47: 702–709. 25985137 10.1038/ng.3285

[64] Lee SH, Goddard ME, Visscher PM, van der Werf JH. Using the realized relationship matrix to disentangle confounding factors for the estimation of genetic variance components of complex traits. Genet Sel Evol. 2010;42: 22. 20546624 10.1186/1297-9686-42-22 PMC2903499

[65] Browning SR, Browning BL. Identity-by-descent-based heritability analysis in the Northern Finland Birth Cohort. Hum Genet. 2013;132: 129–138. 23052944 10.1007/s00439-012-1230-y PMC3543768

[66] Yang J, Zaitlen NA, Goddard ME, Visscher PM, Price AL. Advantages and pitfalls in the application of mixed-model association methods. Nat Genet. 2014;46: 100–106. 24473328 10.1038/ng.2876 PMC3989144

[67] Zhu H, Zhou X. Statistical methods for SNP heritability estimation and partition: A review. Comput Struct Biotechnol J. 2020;18: 1557–1568. 32637052 10.1016/j.csbj.2020.06.011 PMC7330487

[68] Mayhew AJ, Meyre D. Assessing the Heritability of Complex Traits in Humans: Methodological Challenges and Opportunities. Curr Genomics. 2017;18: 332–340. 29081689 10.2174/1389202918666170307161450 PMC5635617

[69] Barry C-JS, Walker VM, Cheesman R, Davey Smith G, Morris TT, Davies NM. How to estimate heritability: a guide for genetic epidemiologists. Int J Epidemiol. 2023;52: 624–632. 36427280 10.1093/ije/dyac224 PMC10114051

[70] Yang J, Benyamin B, McEvoy BP, Gordon S, Henders AK, Nyholt DR, Common SNPs explain a large proportion of the heritability for human height. Nat Genet. 2010;42: 565–569. 20562875 10.1038/ng.608 PMC3232052

[71] Yang J, Lee SH, Goddard ME, Visscher PM. GCTA: a tool for genome-wide complex trait analysis. Am J Hum Genet. 2011;88: 76–82. 21167468 10.1016/j.ajhg.2010.11.011 PMC3014363

[72] Harold D, Abraham R, Hollingworth P, Sims R, Gerrish A, Hamshere ML, Genome-wide association study identifies variants at CLU and PICALM associated with Alzheimer’s disease. Nat Genet. 2009;41: 1088–1093. 19734902 10.1038/ng.440 PMC2845877

[73] Lee SH, Harold D, Nyholt DR, ANZGene Consortium, International Endogene Consortium, Genetic and Environmental Risk for Alzheimer’s disease Consortium, Estimation and partitioning of polygenic variation captured by common SNPs for Alzheimer’s disease, multiple sclerosis and endometriosis. Hum Mol Genet. 2013;22: 832–841. 23193196 10.1093/hmg/dds491 PMC3554206

[74] Naj AC, Jun G, Beecham GW, Wang L-S, Vardarajan BN, Buros J, Common variants at MS4A4/MS4A6E, CD2AP, CD33 and EPHA1 are associated with late-onset Alzheimer’s disease. Nat Genet. 2011;43: 436–441. 21460841 10.1038/ng.801 PMC3090745

[75] Ridge PG, Mukherjee S, Crane PK, Kauwe JSK, Alzheimer’s Disease Genetics Consortium. Alzheimer’s disease: analyzing the missing heritability. PLoS One. 2013;8: e79771. 24244562 10.1371/journal.pone.0079771 PMC3820606

[76] Ridge PG, Hoyt KB, Boehme K, Mukherjee S, Crane PK, Haines JL, Assessment of the genetic variance of late-onset Alzheimer’s disease. Neurobiol Aging. 2016;41: 200.e13–200.e20. 10.1016/j.neurobiolaging.2016.02.024 PMC4948179 27036079

[77] Lo M-T, Kauppi K, Fan C-C, Sanyal N, Reas ET, Sundar VS, Identification of genetic heterogeneity of Alzheimer’s disease across age. Neurobiol Aging. 2019;84: 243.e1–243.e9. 10.1016/j.neurobiolaging.2019.02.022 PMC6783343 30979435

[78] Nazarian A, Kulminski AM. Evaluation of the Genetic Variance of Alzheimer’s Disease Explained by the Disease-Associated Chromosomal Regions. J Alzheimers Dis. 2019;70: 907–915. 31282417 10.3233/JAD-190168 PMC7243481

[79] Wang H, Lo M-T, Rosenthal SB, Makowski C, Andreassen OA, Salem RM, Similar Genetic Architecture of Alzheimer’s Disease and Differential APOE Effect Between Sexes. Front Aging Neurosci. 2021;13: 674318. 34122051 10.3389/fnagi.2021.674318 PMC8194397

[80] Baker E, Leonenko G, Schmidt KM, Hill M, Myers AJ, Shoai M, What does heritability of Alzheimer’s disease represent? PLoS One. 2023;18: e0281440. 37115753 10.1371/journal.pone.0281440 PMC10146480

[81] International HapMap Consortium. The International HapMap Project. Nature. 2003;426: 789–796. 14685227 10.1038/nature02168

[82] 1000 Genomes Project Consortium, Abecasis GR, Auton A, Brooks LD, DePristo MA, Durbin RM, An integrated map of genetic variation from 1,092 human genomes. Nature. 2012;491: 56–65. 23128226 10.1038/nature11632 PMC3498066

[83] Abegaz F, Chaichoompu K, Génin E, Fardo DW, König IR, Mahachie John JM, Principals about principal components in statistical genetics. Brief Bioinform. 2019;20: 2200–2216. 30219892 10.1093/bib/bby081

[84] Beach TG, Monsell SE, Phillips LE, Kukull W. Accuracy of the clinical diagnosis of Alzheimer disease at National Institute on Aging Alzheimer Disease Centers, 2005–2010. J Neuropathol Exp Neurol. 2012;71: 266–273. 22437338 10.1097/NEN.0b013e31824b211b PMC3331862

[85] Bulik-Sullivan B, Finucane HK, Anttila V, Gusev A, Day FR, Loh P-R, An atlas of genetic correlations across human diseases and traits. Nat Genet. 2015;47: 1236–1241. 26414676 10.1038/ng.3406 PMC4797329

[86] Bulik-Sullivan BK, Loh P-R, Finucane HK, Ripke S, Yang J, Schizophrenia Working Group of the Psychiatric Genomics Consortium, LD Score regression distinguishes confounding from polygenicity in genome-wide association studies. Nat Genet. 2015;47: 291–295. 25642630 10.1038/ng.3211 PMC4495769

[87] Evans LM, Tahmasbi R, Vrieze SI, Abecasis GR, Das S, Gazal S, Comparison of methods that use whole genome data to estimate the heritability and genetic architecture of complex traits. Nat Genet. 2018;50: 737–745. 29700474 10.1038/s41588-018-0108-x PMC5934350

[88] Zheng J, Erzurumluoglu AM, Elsworth BL, Kemp JP, Howe L, Haycock PC, LD Hub: a centralized database and web interface to perform LD score regression that maximizes the potential of summary level GWAS data for SNP heritability and genetic correlation analysis. Bioinformatics. 2017;33: 272–279. 27663502 10.1093/bioinformatics/btw613 PMC5542030

[89] Brainstorm Consortium, Anttila V, Bulik-Sullivan B, Finucane HK, Walters RK, Bras J, Analysis of shared heritability in common disorders of the brain. Science. 2018;360. doi:10.1126/science.aap8757 PMC6097237 29930110

[90] Chen H-H, Petty LE, Sha J, Zhao Y, Kuzma A, Valladares O, Genetically regulated expression in late-onset Alzheimer’s disease implicates risk genes within known and novel loci. Transl Psychiatry. 2021;11: 618. 34873149 10.1038/s41398-021-01677-0 PMC8648734

[91] Jansen IE, Savage JE, Watanabe K, Bryois J, Williams DM, Steinberg S, Genome-wide meta-analysis identifies new loci and functional pathways influencing Alzheimer’s disease risk. Nat Genet. 2019;51: 404–413. 30617256 10.1038/s41588-018-0311-9 PMC6836675

[92] Monereo-Sánchez J, Schram MT, Frei O, O’Connell K, Shadrin AA, Smeland OB, Genetic Overlap Between Alzheimer’s Disease and Depression Mapped Onto the Brain. Front Neurosci. 2021;15: 653130. 34290577 10.3389/fnins.2021.653130 PMC8288283

[93] Wu P, Du B, Wang B, Yin R, Lv X, Dai Y, Joint Analysis of Genome-Wide Association Data Reveals No Genetic Correlations Between Low Back Pain and Neurodegenerative Diseases. Front Genet. 2021;12: 744299. 34630533 10.3389/fgene.2021.744299 PMC8493037

[94] de la Fuente J, Grotzinger AD, Marioni RE, Nivard MG, Tucker-Drob EM. Integrated analysis of direct and proxy genome wide association studies highlights polygenicity of Alzheimer’s disease outside of the APOE region. PLoS Genet. 2022;18: e1010208. 35658006 10.1371/journal.pgen.1010208 PMC9200312

[95] Marioni RE, Harris SE, Zhang Q, McRae AF, Hagenaars SP, Hill WD, GWAS on family history of Alzheimer’s disease. Transl Psychiatry. 2018;8: 99. 29777097 10.1038/s41398-018-0150-6 PMC5959890

[96] Robertson A, Lerner IM. The Heritability of All-or-None Traits: Viability of Poultry. Genetics. 1949;34: 395–411. 17247323 10.1093/genetics/34.4.395 PMC1209454

[97] Lee SH, Wray NR, Goddard ME, Visscher PM. Estimating missing heritability for disease from genome-wide association studies. Am J Hum Genet. 2011;88: 294–305. 21376301 10.1016/j.ajhg.2011.02.002 PMC3059431

[98] Zhang Q, Sidorenko J, Couvy-Duchesne B, Marioni RE, Wright MJ, Goate AM, Risk prediction of late-onset Alzheimer’s disease implies an oligogenic architecture. Nat Commun. 2020;11: 4799. 32968074 10.1038/s41467-020-18534-1 PMC7511365

[99] Escott-Price V, Hardy J. Genome-wide association studies for Alzheimer’s disease: bigger is not always better. Brain Commun. 2022;4: fcac125. 35663382 10.1093/braincomms/fcac125 PMC9155614

[100] Lai D, Zhang M, Li R, Zhang C, Zhang P, Liu Y, Identifying Genes Associated with Alzheimer’s Disease Using Gene-Based Polygenic Risk Score. J Alzheimers Dis. 2023;96: 1639–1649. 38007651 10.3233/JAD-230510 PMC11154163

[101] Speed D, Cai N, UCLEB Consortium, Johnson MR, Nejentsev S, Balding DJ. Reevaluation of SNP heritability in complex human traits. Nat Genet. 2017;49: 986–992. 28530675 10.1038/ng.3865 PMC5493198

[102] Speed D, Balding DJ. SumHer better estimates the SNP heritability of complex traits from summary statistics. Nat Genet. 2019;51: 277–284. 30510236 10.1038/s41588-018-0279-5 PMC6485398

[103] Sirugo G, Williams SM, Tishkoff SA. The missing diversity in human genetic studies. Cell. 2019;177: 26–31. 30901543 10.1016/j.cell.2019.02.048 PMC7380073

[104] Eichler EE, Flint J, Gibson G, Kong A, Leal SM, Moore JH, Missing heritability and strategies for finding the underlying causes of complex disease. Nat Rev Genet. 2010;11: 446–450. 20479774 10.1038/nrg2809 PMC2942068

[105] Yang J, Bakshi A, Zhu Z, Hemani G, Vinkhuyzen AAE, Nolte IM, Genome-wide genetic homogeneity between sexes and populations for human height and body mass index. Hum Mol Genet. 2015;24: 7445–7449. 26494901 10.1093/hmg/ddv443

[106] Luo Y, Li X, Wang X, Gazal S, Mercader JM, 23 and Me Research Team, Estimating heritability and its enrichment in tissue-specific gene sets in admixed populations. Hum Mol Genet. 2021;30: 1521–1534. 33987664 10.1093/hmg/ddab130 PMC8330913

[107] Purcell S. Variance components models for gene–environment interaction in twin analysis. Twin Res. 2002;5: 554–571. 12573187 10.1375/136905202762342026

[108] Zuk O, Hechter E, Sunyaev SR, Lander ES. The mystery of missing heritability: Genetic interactions create phantom heritability. Proc Natl Acad Sci U S A. 2012;109: 1193–1198. 22223662 10.1073/pnas.1119675109 PMC3268279

[109] Felson J. What can we learn from twin studies? A comprehensive evaluation of the equal environments assumption. Soc Sci Res. 2014;43: 184–199. 24267761 10.1016/j.ssresearch.2013.10.004

